# High-protein diets increase microbiota associated *p-*cresol production in the colon and reduce gut barrier function in a sex-dependent manner

**DOI:** 10.1007/s00394-025-03885-6

**Published:** 2026-02-12

**Authors:** Daniel James, Maria Batool, Carlos Poveda, Zeynep Hayirli, Chloe Callow, Munawar Abbas, Brandon Linden, John Gibson, Bruce A. Griffin, J. Stephen Elmore, Gemma E. Walton, M. Denise Robertson, Marie C. Lewis

**Affiliations:** 1 Department of Food and Nutritional Sciences, Whiteknights Campus, University of Reading, Reading, RG6 6DZ UK; 2 APC Microbiome Ireland, University College Cork, Cork, Ireland; 3 Cancer Research @UCC, College of Medicine and Health, University College Cork, Cork, Ireland; 4 Food and Feed Innovations, Newcastle Rd, Wore, N Shropshire, Woodstock, CW3 95N UK; 5 Department of Nutrition, Food & Exercise Sciences, University of Surrey, Guildford, GU2 7XH UK

**Keywords:** Dietary protein, Gut microbiota, Sexual dimorphism, Piglet model, Microbial-derived metabolic end products

## Abstract

Protein is an essential nutrient, but the detrimental effects of excess dietary protein on gut health are often overlooked. Protein fermentation by colonic microbiota may impair barrier function by increasing toxic metabolite production. We previously identified sex-by-protein interactions affecting the microbiota and its metabolites in vitro. Do sex-by-protein interactions in colonic protein fermentation lead to a sexually dimorphic response in gut barrier function in vivo? We hypothesised that high-protein diets would elicit sex-specific effects on microbiota and barrier function. Twenty sibling-matched male (*n* = 10) and female (*n* = 10) piglets were fed high-protein (28%) or standard-protein (SP; 18%) diets for four weeks. Bacterial populations were assessed using 16 S rRNA sequencing, urinary metabolites via SPME/GC-MS, and gut barrier proteins via quantitative fluorescence immunohistology. High-protein diets increased bacteria-derived *p-*cresol and reduced E-cadherin and CD45 + protein expression without altering microbiota composition. Females on high-protein diets had greater abundances of *Staphylococcus* and *Chryseobacterium*, elevated *p-*cresol, and reduced ZO-1 expression compared to males. High-protein diets appear to reduce barrier function and increase protein-associated toxic metabolite production in sexually dimorphic manners in pigs. If these results are replicated in humans, it indicates requirements for sex-specific nutritional strategies.

## Introduction

There is increasing evidence that excessive dietary protein leads to the production of metabolites capable of increasing intestinal permeability in vitro [1–3] and thus, if this occurs in vivo it may permit increased levels of bacteria and microbial-derived products to enter the underlying tissues [4, 5]. Subsequent immune responses to such products could result in chronic systemic inflammation that represents an early origin of prevalent conditions such as metabolic dysfunction-associated steatotic liver disease (MASLD), [6] cardiovascular disease (CVD), [7] and type 2 diabetes (T2D) [8]. However, several current heath recommendations advocate increasing dietary protein intake by up to 100% in an attempt to mitigate the onset and progression of sarcopenia in older adults [9]. Such recommendations would seem to disregard protein intakes in high- and middle-income countries, which often exceed government guidelines of 0.75–0.8 g protein/kg of body weight [10]. Additionally, specific groups, including athletes and bodybuilders, often consume high levels of dietary protein with the aim of improving performance [11, 12]. Despite this, the in vivo impact of excessive dietary protein on gut barrier function remains largely unexplored.

Different bacterial populations have varying propensities for proliferation in high-protein environments, which is dependent upon the protease profile expressed by each population. Consequently, increased protein availability in the colon has the potential to skew the microbiota by favouring species that are most capable of utilising proteins, while potentially disadvantaging the populations of bacteria that preferentially ferment carbohydrates, which are more likely to be beneficial to host health [13]. Additionally, protein fermentation by the microbiota can result in increased production of potentially detrimental metabolites, including ammonia, phenol, indole and *p-*cresol [14, 15]. Previous studies using Caco-2 cell lines have demonstrated that these metabolites impair gut barrier function in vitro [3, 16, 17] by reducing the expression of tight-cell junction (TCJ) proteins responsible for maintaining barrier integrity and limiting paracellular permeability, including zona occludens (ZO-1), occludin, and claudins [3]. However, it is currently unclear whether the increased production of ammonia, *p-*cresol, phenol, and/or indole resulting from microbial protein fermentation in the colon leads directly to gut barrier dysfunction in vivo.

Elevated levels of intestinal permeability enable an incursion of bacteria and bacterial products into the lamina propria, potentially inducing local inflammatory responses. For example, lipopolysaccharide (LPS), an endotoxin from the wall of Gram-negative bacteria, can pass through disrupted TCJs when the gut barrier is compromised [18]. In the lamina propria, LPS is recognised by Toll-like receptor 4 (TLR4) on the surface of some immune-associated cells which causes a downstream signalling cascade and ultimately increase the production of inflammatory cytokines [19]. Pro-inflammatory cytokines bind to receptors on epithelial cells, triggering an increase in myosin light chain kinase (MLCK) production which phosphorylates myosin light chain (MLC), a process that causes actomyosin contraction and further increases transit of microbial derived products through TCJs [20]. If increased protein fermentation in the colon disrupts barrier function in vivo,* via* the production of potentially detrimental metabolites, it could initiate a positive feedback loop which further depletes the gut barrier and perpetuates chronic systemic inflammation.

Sex differences in susceptibility to gut-related conditions and chronic inflammatory diseases is well documented. For example, adult females are more predisposed to developing autoimmune and inflammatory diseases including systemic lupus erythematosus (SLE), multiple sclerosis (MS) [21], Crohn’s disease [22] and irritable bowel syndrome (IBS) [23] compared to males. The sex differences in these conditions are likely influenced by disparities in the immune systems of males and females, with adult females typically producing more robust protective inflammatory responses to foreign antigens and infections than males do [24]. These differences may be partly due to females possessing two X chromosomes which contain many genes encoding various aspects of immune function [25], while males have only one X chromosome. Additionally, the gut microbiota is a key driver for immune development, as demonstrated in germ-free animal models which have severely underdeveloped immune systems until commensal microbes are introduced, at which point immune development commences [26–29]. Furthermore, different bacterial-derived components, including short chain fatty acids (SCFA) have been demonstrated to exert specific effects on diverse components of immunity [30, 31]. A growing body of evidence is consistent with males and females having distinct microbiotas. Females typically have more diverse microbiota compositions than males [32–34] and greater abundances of taxa including Bacillota (Firmicutes), Actinbobacteria, *Bifidobacterium*,* Alistipes* and Odoribacter, whereas males tend to have higher levels of Bacteroidetes, *Fusobacterium*,* Escherichia*,* Clostridium*,* Bacteroides* and *Prevotella* [35–40]. This could be contributing to the observed disparities in immune system responses between the sexes, even in prepubescent animals [41], and also predispositions to immune-related diseases.

It is perhaps unsurprising that since there are sex differences in gut bacterial populations, male and female microbiotas have been reported to respond differently to dietary interventions, including to supplementation with inulin, high amylose wheat and high fat diets [42–45]. Consistent with this, we have previously used in vitro batch culture systems to demonstrate that bacterial fermentation of mycoprotein expanded the population of propionate-producing bacteria, while whey protein significantly enhanced lactobacilli growth more in microbiotas originating from males than from females [46]. These changes in bacterial growth coincided with significantly greater synthesis of phenol following protein fermentation by female microbiota, but higher levels of *p-*cresol production following fermentation by male microbiotas [46]. Furthermore, there are significant innate differences in gut barrier function between males and females. Barrier function is more consistent among females, resulting in minimal variation in intestinal permeability between individuals, while males exhibit broader gut barrier variations [40, 47]. While adult male gut barrier function is more vulnerable to acidosis and hypoxia [48], adult females show greater susceptibility to barrier impairment in response to non-steroidal anti-inflammatory drugs (NSAIDs) compared to males [47]. Given the intricate and reciprocal interactions between the microbiota, gut barrier function and immunity coupled with the sex differences inherent in each of these systems, it follows that sex-dependent dietary responses in the microbiota could result in sex-specific differences in gut barrier disruption in response to increased protein availability in the colon. While, the effects of high protein diets on the microbiota and barrier function have received little attention, as recently reviewed [49], to the best of our knowledge no explorations of whether the effect of excess protein differentially impact males and females have previously been conducted. It is well established that omnivorous pigs share many physiological similarities with humans, including intestinal physiology, microbiota composition and immune system characteristics, and are more outbred than rodent models and thus better reflect human populations [50–54]. Full genome studies show that there are more differences between rodents and humans compared to pigs and humans [55, 56]. In pigs and humans, gut microbiotas are considerably more stable over time than in rodent models and intra-individual variability is reduced in mice compared to that of humans and pigs [57]. Generally, the microbiota of pigs and humans also share similar diversities and dominant phyla, including Bacteroides and Firmicutes [58, 59]. In addition, the macronutrient requirements of pigs aligns closely with those of humans and we have previously shown that the metabolic profiles in pigs, characterised *via* NMR spectroscopy, were qualitatively comparable to those in humans [60]. This is consistent with pigs being valuable intermediates between highly reductionist, mechanistic studies in rodents and epidemiological studies and clinical trials in humans, and are especially valuable as models for human nutrition exploration.

Based on our prior research using an in vitro fermentation system, we demonstrated significant sex-protein interactions influencing microbiota composition and the production of potentially toxic metabolites capable of impairing gut barrier function. It was thus hypothesised that HP diets would directly and adversely affect intestinal barrier function in sex-dependent manners in vivo. However, given that we previously observed significant increases in phenol and ammonia in females, and significant increases in p-cresol in males (all of which have been shown to disrupt gut barrier function) in response to HP in vitro, it is difficult to predict with any certainty the magnitude or direction of sex-linked changes to barrier function we might observe. The aim of this study was to determine whether increased dietary protein was detrimental to gut barrier function *via* modulations of the gut microbiota and subsequent metabolic output, in a sex-dependent manner, using pig models. The findings of this study could inform sex-specific dietary recommendations to reduce barrier disfunction and mitigate the risk of chronic diseases with sex-based differences in prevalence.

## Methods

### Animal model

The animal housing and all experimental procedures were conducted at the Centre for Dairy Research facility (CEDAR) at the University of Reading in compliance with ARRIVE guidelines and under a UK Home Office License (PP0518767). All experiments were approval by the Reading Animal Welfare and Ethical Review Body (AWERB).

**Fig. 1:**
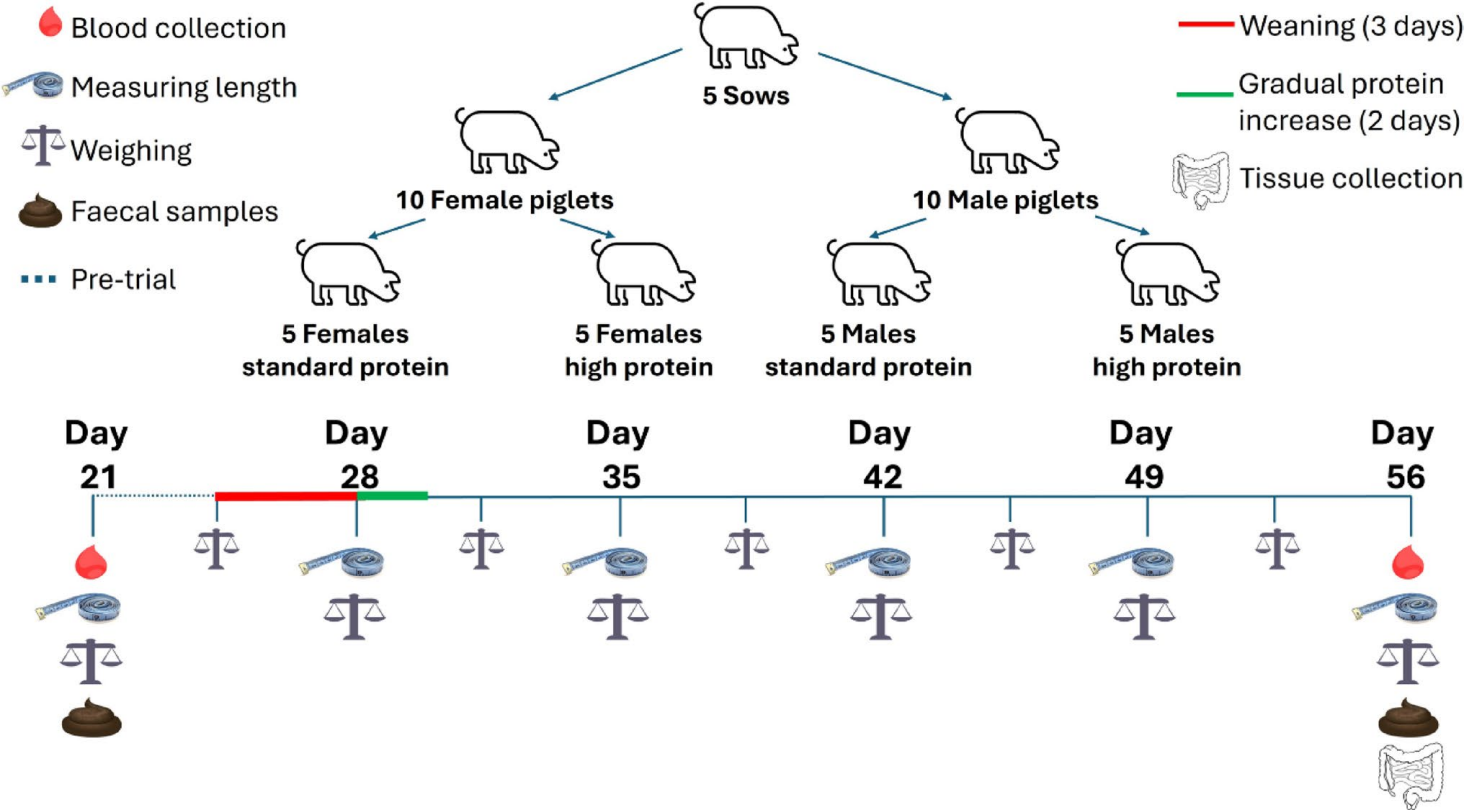
Experimental design used to compare the effect of high protein diets in males and females. At 21 days old, twenty piglets from five sows were brought to the Centre for Dairy Research (CEDAR) at the University of Reading, where they were fed sow replacer milk for three days. This was followed by a gradual transition to standard weaner feed over the following three days. On day 28, piglets were divided into treatment groups matched by litter and balanced by sex and received either standard protein (18% dietary protein) or a high protein (28% dietary protein) diet until they reached 56 days old. Faecal and blood samples were taken pre-weaning (day 21) and at the end of the trail (day 56). Weight and length measurements were taken sequentially through the trial period. At the end of the trial, the piglets were dissected, and colon tissue was snap frozen in liquid nitrogen for future processing

To determine potential sex differences in the effects of HP diets on gut microbiota composition, the production of microbial-derived end-products, intestinal integrity and mucosal immune development, a two-by-two litter-matched pig trial was conducted (Fig. 1). Litter-matching accounts for the genetic variability between groups, enabling studies to be conducted with fewer animals while maintaining statistical power. At three weeks old, 20 healthy Large White F1 hybrid piglets were transported from a commercial indoor pig farm to temperature-controlled indoor facilities at CEDAR. To help reduce the time it took for the piglets to learn to drink from bowls, they were initially housed together to facilitate learning through emulation. This mixing also aided microbial transfer between piglets to reduce the impact of ‘litter’ on gut bacterial populations. After one day of group housing, piglets were individually housed on wood shavings in wire mesh-walled pens to permit social interactions and further bacterial transfer (to reduce the effects of ‘pen’), whilst preventing non-autologous coprophagy and thus ensure tight dietary control. All housing was maintained using stringent biosecurity measures to prevent contamination, including disinfectant foot wells and limited personnel access. For the first three days after arrival at CEDAR, the piglets were fed commercial sow milk replacer (Table 1 Target Feeds, Whitchurch, UK) and to limit the potential for post-weaning diarrhoea, piglets were transitioned onto commercial weaner mash containing 18% protein (Table 2, Target Feeds, Whitchurch, UK) over the following three days. The 18% dietary protein content for the SP group was chosen because it is approximately the habitual dietary intake found in humans, and it is the lowest limit of protein recommended for piglets while maintaining good overall health. On the seventh day after arriving at CEDAR, male and female sibling piglets were litter-matched into treatment groups with half of both the sexes receiving an additional 10% protein composed of equal parts derived from milk (BaltMilk, Kaunas, Lithuania) soya (Wilmar internation, Singapore), pea (Wilmar internation, Singapore) and fish which were mixed into their regular mash at each feed. These sources of protein were chosen based on results from our previous in vitro trial which showed they produced substantial changes to the microbiota and its metabolites [46]. All other total dietary macronutrient intakes were kept the same between groups to avoid the confounding impact of displacing nutrients. This resulted in five males and five females receiving diets comprising 18% protein (SP group) which acted as the control group, while five of their male and five of their female siblings consumed diets with 28% protein (HP group), resulting in *n* = 5/sex/treatment group in total. This complies with the power calculations we conducted based on our previous similar pig trials.

**Table 1:** Composition of Sow milk replacer

Ingredients	Percentage (%)
Crude oil	20
Crude protein	
Full cream milk & whey concentrate	15
Crude fibre	0
Ash	3.5
Sodium	0.2
Lactose	50
Lysine	1.45
Methionine	0.6

Calculated as % of total feed

**Table 2:** Composition of the weaner feed. Calculated as % of total feed; HP diets included an additional 10% protein provided as a supplement, but the baseline diets contained 18% protein and were consumed by all piglets

Ingredients (%)	Standard protein	High protein
Barley	6	5.4
Oats	15	13.5
Wheat		
Raw ground	15	13.5
Micro ground	15	13.5
Maize	15	13.5
Whey powder	7.5	6.75
Soya Hipro	8.25	7.425
Full fat soya masham	5	4.5
Fishmeal	5	4.5
L Lysine	0.1	0.09
Soya oil	5	4.5
Limestone flour	0.25	0.225
Dibasic calcium phosphate flour	2	1.8
Salt	0.4	0.36
Weaner Trials PMX. 140	0.5	0.45
Milk protein	–	2.5
Pea protein	–	2.5
Fish protein	–	2.5
Soya protein	–	2.5

Vitamin and micronutrient content of pig feed mix: copper, 146 mg/kg; calcium, 1 mg/kg; phosphate, 0.8 mg/kg; AV-phosphate, 0.62 mg/kg; salt, 0.8 mg/kg; sodium, 0.32 mg/kg; vitamin A, 12 mg/kg; vitamin D, 2 mg/kg; vitamin E, 250 mg/kg; tryptophan, 0.21 mg/kg; threonine, 0.67 mg/kg; methionine, 0.32 mg/kg; T-lysine, 1.04 mg/kg; AV-lysine, 0.94 mg/kg; ash, 6 g/kg; fibre, 17.5 g/kg; oil, 9.2 g/kg

Fresh faeces and blood (*via* venepuncture) were collected from day 0 of the trial (when piglets were three weeks old) and at the end of the trial (day 56) at eight weeks old. Faecal collection consisted of observing the piglets and collecting faeces immediately upon defecation, in order to prevent contamination. Upon completion of the trial at 56 days, piglets were given a sedative (Stresnil^®^, Elanco, US) followed by an overdose of barbiturate (Euthatal^®^, Vetoquinol, UK). During postmortem, rectal contents, urine (collected directly from the bladder using a needle and syringe) and snap frozen in liquid nitrogen. Descending colon tissue samples were collected and immediately mounted in Optimal cutting temperature (OCT) mounting medium (Cell Path, UK) and frozen in 2-methylbutane (Fisher Scientific Ltd, UK) over liquid nitrogen. All samples were stored at −80 °C for future analysis. Piglets were blood sampled, fed and euthanized, and samples analysed, in a random order to prevent treatment group bias.

## Bacterial DNA isolation

DNA was isolated from faecal samples using the QIAmp DNA PowerFaecal Kit (Qiagen, Hilden, Germany) according to the manufacturer’s instructions. Briefly, 250 mg of faeces was diluted in 800 µL of solution A lysis buffer (CD1) in a 2 mL Eppendorf and vortexed for 10 min to ensure thorough mixing. After centrifugation (15,000 g for 1 min), the supernatant was treated sequentially with CD2 (precipitation solution) and CD3 (binding buffer), with intermediate centrifugation steps. The lysate was loaded onto MB Spin Columns, washed with washing buffers, and eluted with solution C6, an elution buffer. The resulting DNA was stored at -20 °C for downstream analysis.

## PCR, 16 S rRNA sequencing and library Preparation

The isolated bacterial DNA was sent to Novogene Ltd (Cambridge, UK) for 16 S rRNA gene sequencing. The DNA underwent PCR amplification of the V4 and V5 regions of the 16 S rRNA gene using the primer pairs 519 F (GTGCCAGCMGCCGCGGTAA) and 907R (CCGTCAATTCCTTTGAGTTT). All PCR reactions were carried out with 15 µL of Phusion^®^ High-Fidelity PCR Master Mix (New England Biolabs); 0.2 µM of forward and reverse primers and 10 ng template DNA. Thermal cycling of initial denaturation at 98 °C for 1 min, followed by 30 cycles of denaturation at 98 °C for 10 s, primer annealing at 50 °C for 30 s, and elongation at 72 °C for 30 s followed by a final 72 °C elongation step for 5 min.

Sequencing libraries were generated using the NEB Next^®^ Ultra™ II FS DNA PCR-free Library Prep Kit (New England Biolabs, USA), adhering to the manufacturer’s recommendations, and indices were added to each sample. Library quality was assessed using the Qubit fluorometer and real-time PCR for quantification, and the bioanalyser was used to check the size distribution of the libraries. The quantified libraries were then pooled and sequenced using the Illumina platform.

## Bioinformatics analysis

Sequences were demultiplexed by Novogene Ltd. before being returned for detailed analysis. Using QIIME2 [61], base pairs were trimmed based on sequence quality. Following this, pre-filtering of sequence contaminants, removal of chimeras, merging of paired end-reads and data denoising was conducted using the DADA2 pipeline [62] within QIIME2. After completing quality filtering steps, we identified 33, 991 ASVs across *n* = 40 samples. The summary statistics for reads aligned per sample to these ASVs are as follows: 1st Quartile: 59,008, Median: 63,074, Mean: 63,030.93, 3rd Quartile: 67,935, Max: 89,139. The SILVA 138 [63] full-sequence classifier for the V4 and V5 regions was trained based on the minimum and maximum sequence lengths of samples and used to classify sequences into their specific ASVs.

Diversity analyses were conducted using QIIME2 for core phylogenetic differences. Microbial alpha diversity was assessed using Faith’s phylogenetic diversity (PD) [64], observed features, and Shannon diversity. Beta diversity was evaluated through weighted UniFrac and Bray-Curtis distance measurements, and principal coordinates analysis (PCoA) was conducted to visualise differences between treatment groups. Differences in normalised bacterial expression between groups were assessed by DESeq2 (v1.4.2) [65], with a significance threshold of *p* < 0.05. Predictive modelling of microbiota function based on 16 S data was executed using PICRUSt2 on the R library ggpicrust2 (v2) [66], which generated outputs for MetaCyc [67] (total identified: 426), Keoto Encyclopaedia of Genes and Genomes (KEGG) pathways [68] (total identified: 264) and KEGG orthologs (KOs) (total identified: 7532). The KOs and KEGG pathways were identified and labelled using R packaged KEGGREST [69]. Differential abundance analyses of KOs, MetaCyc and KEGG pathways was conducted using DESeq2. All figures were made using ggplot2 [70]. 

## SPME-GC/MS for urinary metabolite analysis

Volatile compound characterisation of urine samples was performed using an automated headspace solid-phase microextraction (SPME) technique connected to gas chromatographs-mass spectrometry (GC-MS). The equipment included an Agilent 110 PAL injection system, an Agilent 7890 GC, and a 5975 mass-selective detector (MSD). The SPME fibre used had a 75 μm coating of divinylbenzene/Carboxen™ on polydimethylsiloxane (Supelco, Bellefonte, PA). One mL of each sample was place in the system, equilibrated for 10 min at 35 °C, and then extracted for 30 min while being agitated at 500 rpm (5 s on, 2 s off). Post-extraction, the analytes were desorbed in splitless mode into a Zebron ZB5MS fused silica capillary column (30 m x 0.25 mm i.d., 1 μm thickness; Phenomenex Torrance, CA), with the splitter opening after 0.75 min (100:1 split). Both the fibre desorption and the GC program began simultaneously. The GC oven temperature started at 60 °C, ramped up at a rate of 5 °C/min to 260 °C, and held for 1 min. Helium served as the carrier gas at a flow of 0.9 mL/min.

The mass spectrometer was set to electron impact mode at an electron energy of 70 eV, scanning from m/z 20 to m/z 280 at a rate of 1.9 scans per second. Compound identification was performed using a spectral library, and relative concentrations were calculated by comparing peak areas to a 1 µL internal standard of 1,2-dichlorobenzene at 10 mg/L. Compound identification was performed by library search and identifications were confirmed by running reference compounds under the same chromatographic conditions. The results were later corrected based on urinary osmolality as measured by an OSMOCHECK refractometer (Vitech Scientific, UK).

### Ammonia assay

Urinary ammonia concentrations were measured using an assay kit (Abcam, UK), according to the manufacturer’s instructions. Briefly, urine samples were diluted 100-fold with the assay buffer, and 50 µL of each diluted sample was added to the wells of a microplate, along with the reaction mix, which was a combination of buffer, OxiRed probe and enzyme mix. The plate was then incubated for 60 min at 37 °C in the dark. Measurements were immediately taken using an automatic microplate reader (Tecan, UK) at an optical density of 570 nm. A standard curve generated from the provided standards was used to quantify the ammonia levels in the samples. The ammonia levels were then normalised based on urinary osmolality, measured by an OSMOCHECK (Vitech Scientific, UK).

## Enzyme-linked immunosorbent assays (ELISA)

The concentrations of interleukin (IL)-6 (ThermoFisher Scientific, United States) and lipopolysaccharide (LPS) (AMS Biotechnology, UK) in the plasma of each of the piglets were analysed using ELISA assay kits. The manufacturer’s instructions were followed for both kits and an automatic microplate reader (Tecan, UK) was used to for measuring the optical density at 450 nm. Concentrations of IL-6 and LPS were determined based on the calibration curve of the relative standards.

## Fluorescence immunohistology

Frozen colon tissue sections were cut to 5 μm using a cryostat (OTF500/HS, Bright Instruments, UK). After sectioning, the samples were air-dried for 12 h and then fixed in acetone for 20 min. Non-specific binding sites were blocked using 5% pig serum (Fisher Scientific, UK) and 5% goat serum (Serotec, UK). The sections were then stained using two distinct combinations of monoclonal antibodies. To analyse gut barrier integrity, 50 µL of a mixture of the monoclonal antibodies, mouse anti-human ZO-1 IgG_1_ (clone 1A12, ThermoFisher Scientific, UK; 1/200 concentration) and rabbit anti-pig E-cadherin IgG (clone AA 375–631, Antibodies Online, US; 1/200 concentration), were incubated on the tissue sections overnight at 4 °C. The binding of these antibodies was detected using Goat anti-mouse IgG1 TRITC (Southern Biotechnology, UK; 1/100 concentration) and goat anti-rabbit IgG AF633 (Southern Biotechnology, UK; 1/100 concentration) respectively. Similarly, for the analysis of immune development in the lamina propria the following monoclonal antibodies were used: Mouse anti-pig MHC class II IgG_2a_ (clone MSA3, Cambridge Bioscience; 1/20 concentration), mouse anti-pig CD45 IgG_1_ (clone K25 IE4, ThermoFisher Scientific, UK; 1/10 concentration), mouse anti-pig CD172a IgG_2b_ (clone 74-22-15, Bio-Rad, UK; 1/10 concentration), and mouse anti-pig capillary endothelial cells (IgE, clone MIL11, Bio-Rad, UK; 1/10 concentration). The MIL11 antibody recognises the majority of capillary endothelium cells in the pig gut. An aliquot of 50 µL of this combination of antibodies was incubated on each tissue section at 4 °C overnight. To detect the binding of these antibodies, 50 µL of the following antibody mixture was used: goat anti-mouse IgG_2a_ (ThermoFisher Scientific, UK; 1/100 concentration), goat anti-mouse Ig_G1_ (ThermoFisher Scientific, UK; 1/100 concentration), goat anti-mouse IgG_2a_ (ThermoFisher Scientific, UK; 1/100 concentration) and goat anti-mouse IgE (ThermoFisher Scientific, UK; 1/100 concentration). The following filters were used to ensure no overlap occurred between the channels: C-FL Epi-Fl Filter Blocks DAPI (Excitation Filter EX340-380; dichroic mirror DM400; barrier filter BA435-485), FITC (Excitation filter EX465-495; dichroic mirror DM505; barrier filter BA515-555), TRIT (Excitation filter EX540/25; dichroic mirror DM565; barrier filter BA605/55) and Cy5 (Excitation filter EX620/60x; dichroic mirror DM660; barrier filter BA700/75m).

### Image analysis

The quantification of immunofluorescent staining and co-staining of ZO-1, E-cadherin, and also MHC class II, CD45, MIL11 and CD172a was performed with an in-house macro within ImageJ software version 1.54 [71]. A series of 10 images taken in 16-bit grayscale were systematically acquired along the length of the section of colon for both antibody combinations. This process resulted in the collection of 400 total images (100 representational images for both diet treatments and 50 images for all sex/treatment combinations) captured using a Nikon ECLIPSE Ci fluorescence microscope (Nikon, Japan), equipped with the necessary fluorescence filters. Thresholds for positive and negative pixels were established for each fluorochrome across all images. The number of positive pixels for each fluorochrome combinations was then calculated and expressed as a proportion of the total pixels within the designated area. Normal distribution of the proportion of pixels was achieved with log_10_ transformation and the results are presented as log_10_ proportion of positive pixels. This approach facilitated the quantification of the antigenic markers identified by the primary antibodies, a process corroborated by the findings of Inman et al. [71]

### Statistical analysis

Physical body measurements and metabolomic data were analysed using R [72] on the RStudio [73] (version 4.3.2) environment. Data were first tested for normality using Shapiro-Wilk normality test. The data were non-normally distributed and were therefore subjected to Kruskal-Wallis tests with Wilcoxon Rank-Sum pairwise comparisons. Adjustments for multiple testing were made using the Benjamini-Hochberg method. Demultiplexed microbiome data was process using QIIME2 [61], to trim base pairs, assess sequence quality, filter for contaminants, remove chimeras, and merge paired-end reads, using the DADA2 pipeline [62]. QIIME2 was also used to conduct the PERMANOVA analysis for beta-diversity as well as each alpha diversity analysis. Following this, all further bioinformatic analyses including DESeq2, PICRUSt2 and correlations between microbiome data and metabolites, LPS, IL-6 and TCJ proteins were conducted using R [72] in RStudio [73](version 4.3.2).

For overall model comparisons of protein expression, quantified by fluorescence immunohistology, general linear models (GLMs) on SPSS and Amos (version 27) were used and ‘piglet’ was used as the experimental unit. When significant main effects were identified within the GLMs, post-hoc analyses were conducted with least significant difference (LSD) correction for multiple testing. All data points generated by the trial have been included in the analysis.

## Results

### Weight and length changes associated with high protein diets

**Fig. 2:**
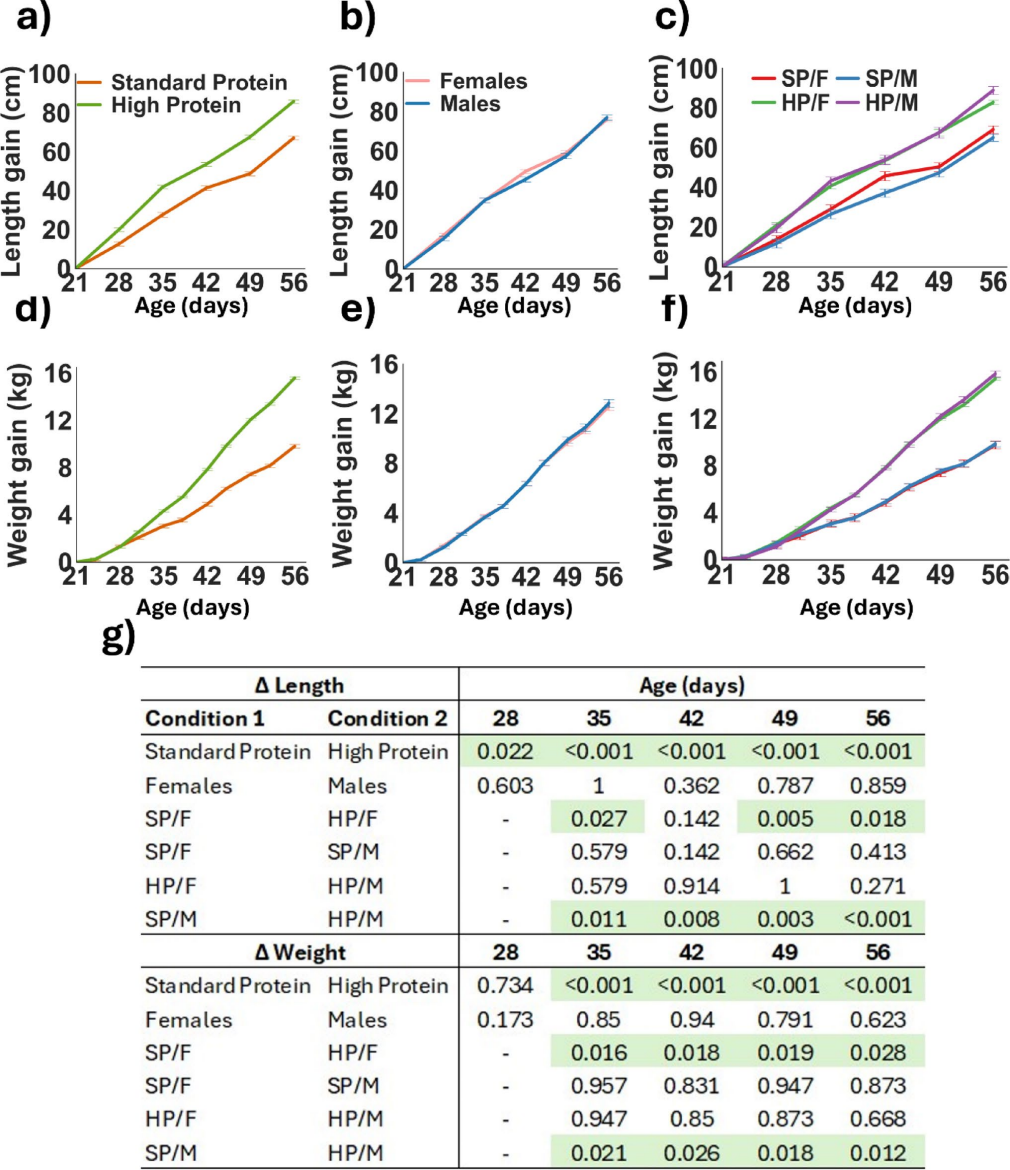
Twenty piglets entered the centre for dairy research (CEDAR) at the University of Reading at 21 days old and fed sow replacer milk for three days before being gradually weaned onto standard weaner feed over the following three days. At 28 days old, the piglets were assigned into litter-matched and sex-balanced treatment groups and fed either a standard protein (SP; 18% dietary protein) or high protein (HP; 28% dietary protein) diet until 56 days old. Increases in body length were tracked to assess the effects of dietary protein **a**, sex **b** and the interaction between sex and protein: standard protein females (SP/F), high protein females (HP/F), standard protein males (SP/M) and high protein males (HP/M) **c**. Similarly, weight gain was measured to evaluate the influence of dietary protein **d**, sex **e**, and the interaction between sex and protein **f**. Significant differences between groups in changes from baseline were determined using the Kruskal-Wallis test with pairwise comparisons and Benjamini Hochberg corrections **g**. Results are presented as mean ± SEM and *n* = 5 piglets/sex/treatment group

The twenty male and female piglets consuming a SP or HP diet were monitored weekly for changes in length and comparisons were made between protein group (Fig. 2a), sex (Fig. 2b), and protein group by sex (Fig. 2c). The changes in weight were measured bi-weekly and compared between SP and HP groups (Fig. 2d), males and females (Fig. 2e) and between the protein and sex interactions (Fig. 2f). Significant differences in length between the HP and SP groups started to appear at 28 days (*p* = 0.02), with weight differences emerging by 31 days of age (*p* < 0.001), persisting for the duration of the study (Fig. 2g). Differences in weight and length were observed exclusively under different protein conditions, with no significant differences between males and females or any interaction effects between sex and protein.

### Increased dietary protein intake influences alpha diversity but not beta diversity

**Fig. 3:**
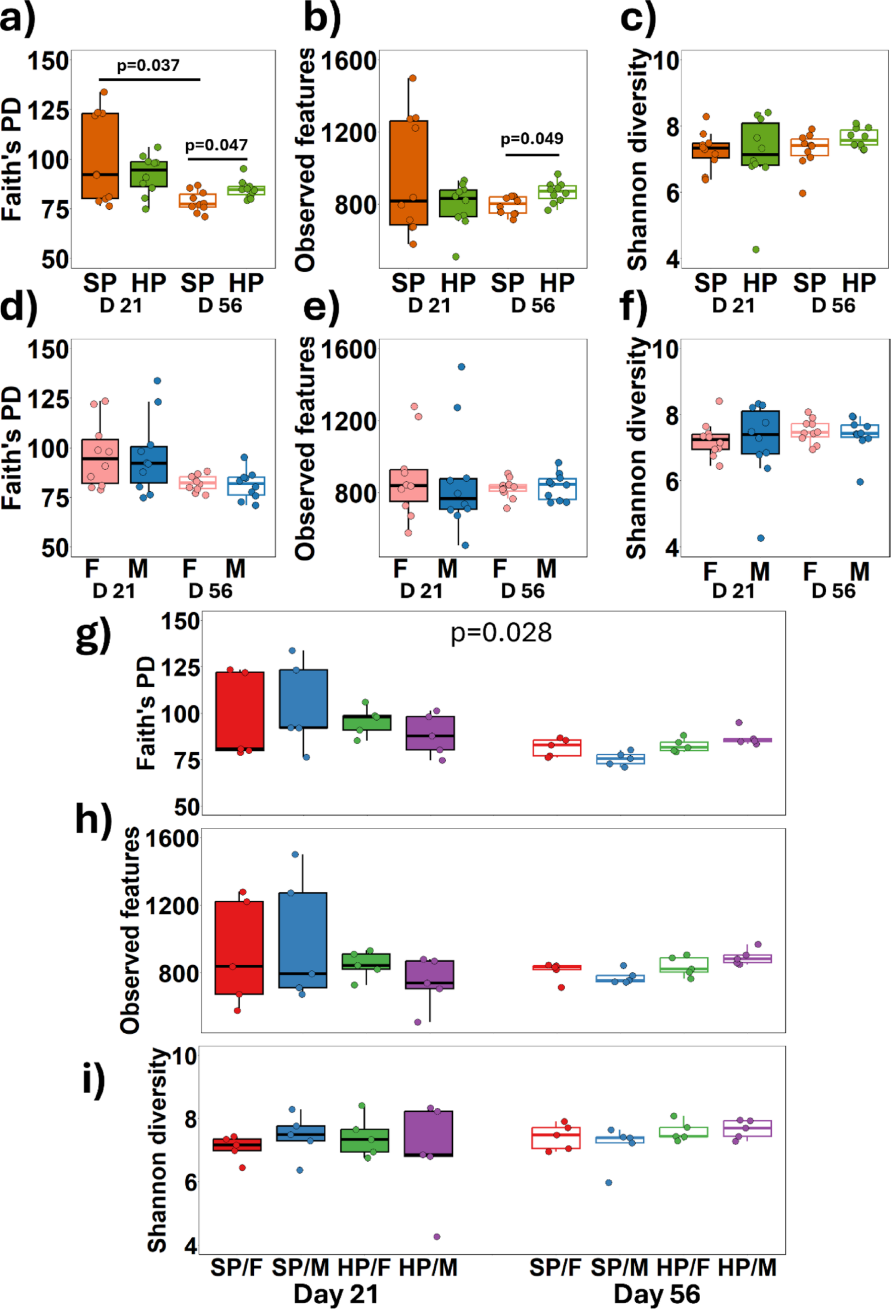
Twenty piglets were allocated into four litter-matched and sex-balanced treatment groups to consume either a standard protein (SP; 18% dietary protein) or high protein (HP; 28% dietary protein) diet for 28 days from 28 days old. Faecal samples were analysed by 16 S rRNA sequencing and the differences in alpha diversity between the HP and SP treatment groups at day 21 and 28 was assessed using Faith’s PD **a**, Observed features **b**, and Shannon diversity **c**. Similarly, differences in alpha diversity between males (M) and females (F) at days 21 and 56 irrespective of diet were measured by Faith’s PD **d**, Observed features **e**, and Shannon diversity **f**. The interaction between sex and diet on alpha diversity was also measured by Faith’s PD **g**, Observed features **h**, and Shannon diversity **i**. Statistical analyses were conducted using Kruskal-Wallis test with pairwise comparisons. Corrections for multiple testing were conducted using the Benjamini-Hochberg method

To investigate the impact of dietary protein on microbiota diversity, alpha and beta diversity analyses were conducted on faecal samples from piglets subjected to high or SP diets over a four-week period. Faith’s PD remained stable in the HP group throughout the 5 weeks, while it significantly declined in the SP group (*p* = 0.037), resulting in lower diversity in the SP group than the HP group by the end of the trial (*p* = 0.047) (Fig. 3a). By day 56, the HP group also exhibited significantly more observed features compared to the SP groups (*p* = 0.049) (Fig. 3b). However, no significant differences between the protein groups were observed for Shannon diversity (*p* = 0.36) (Fig. 3c). There were no differences in any of the measured alpha diversity indices between males and females at any time point (Fig. 3d, e and f). A significant interaction effect between protein intake, sex, and day was detected for Faith’s PD (*p* = 0.028) (Fig. 3g), however, no significant differences emerged in pairwise comparisons after FDR correction. Additionally, no interactions were found between protein intake, sex, and day for observed features (Fig. 3h) or Shannon diversity (Fig. 3i).

**Fig. 4:**
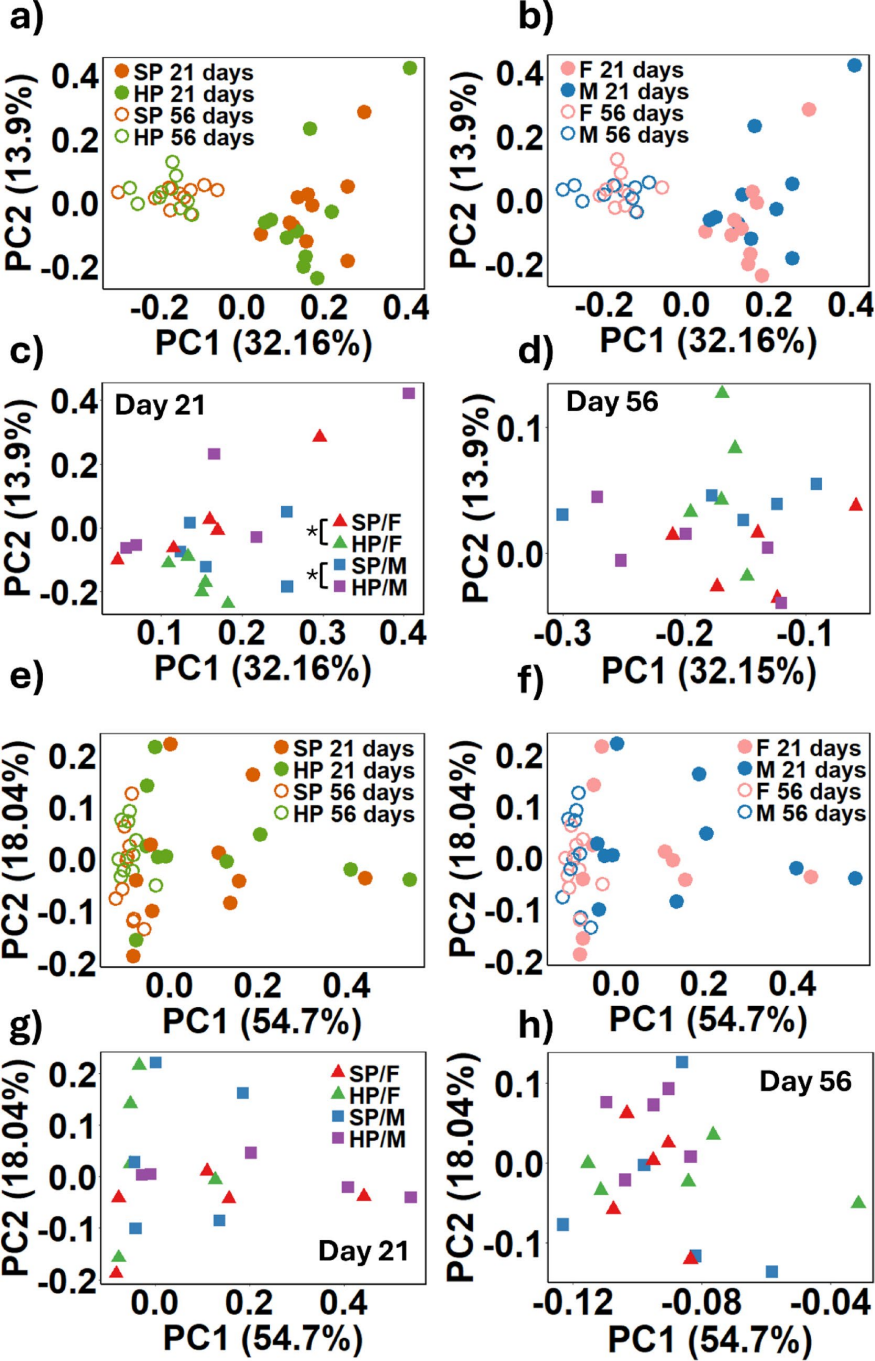
The beta diversity of the faecal microbiota from female (F) (*n* = 10) and male (M) (*n* = 10) piglets consuming either a standard protein (SP; 18% dietary protein) or high protein (HP; 28% dietary protein) in a 2 × 2 model for 28 days. The data is presented as a weighted unifrac distance matrix comparing protein groups at days 21 and 56 **a**, sex at days 21 and 56 **b**, the interaction between protein and sex at day 21 **c** and day 56 **d**. The Bray-Curtis dissimilarity index was also used to compare SP and HP at days 21 and 56 **e**, males and females at days 21 and 56 **f**, the interaction between sex and diet at day 21 **g** and day 56 **h**. The p values were determined using PERMANOVA with pairwise comparison and Benjamini-Hochberg corrections. * = *p* < 0.05

Weighted UniFrac analysis revealed distinct clustering patterns over the trial duration for each group but showed no variations attributable to dietary protein intake or sex (Fig. 4a and b). At the study’s onset (day 21), there were notable differences between HP/F and SP/F (*p* = 0.04), and between HP/F and HP/M (*p* = 0.02) (Fig. 4c). By day 56, these initial differences had dissipated (Fig. 4d). The Bray-Curtis dissimilarity analysis did not identify any significant differences between protein groups (Fig. 4e), males and females (Fig. 4f), or any sex-by-protein interactions at day 21 (Fig. 4g) or day 56 (Fig. 4h).

### Interaction between dietary protein intake and sex on microbiota composition and function

**Fig. 5:**
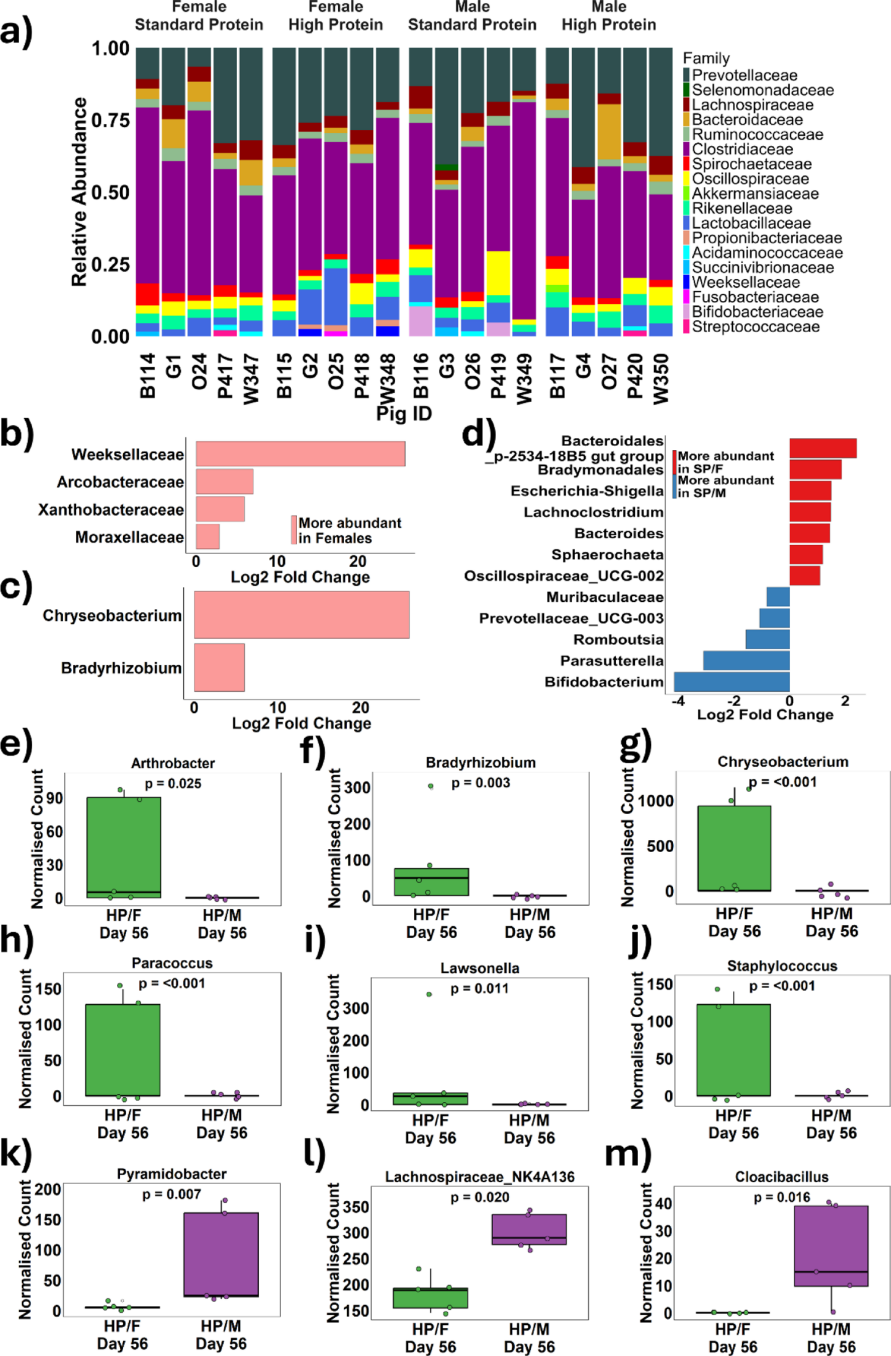
Twenty piglets were litter-matched into either a standard protein (SP; 18% dietary protein) or a high protein (HP; 28% dietary protein) diet for 28 days in a 2 × 2 sex-balanced experimental protocol. The faecal microbiota of the piglets was analysed by 16 S rRNA sequencing to determine the relative abundance of bacterial families after 28 days on their respective diets **a**. Differential abundance analysis was performed using DESeq2 to determine log2 fold changes in bacteria families **b** and genera **c** between males and females. Additionally, differences in genera were analysed between standard protein-fed females (SP/F) and males (SP/M) **d**. Significant differences between high protein-fed males (HP/M) and females (HP/F) were determined using DESeq2 **e**. Multiple testing corrections were applied using the Benjamini-Hochberg method, with significance defined as an adjusted* p*-value < 0.05. Differential abundance analysis was performed using DESeq2 to determine log2 fold changes in bacteria families **b** and genera **c** between males and females. Additionally, differences in genera were analysed between standard protein-fed females (SP/F) and males (SP/M) **d**. Differences between high protein-fed males (HP/M) and females (HP/F) were determined using DESeq2 and those that reached significance are presented: Arthrobacter **e**, Bradyrhizobium **f**, Chryseobacterium **g**, Paracoccus **h**, Lawsonella **i**, Staphylococcus **j**, Pyramidobacter **k**, Lachnospiraceae_NK4A136 **l** and Cloacibacillus **m**. Multiple testing corrections were applied using the Benjamini-Hochberg method, with significance defined as an adjusted p-value < 0.05

Faecal microbiota from piglets that consumed a SP diet or HP diet for four weeks were taxonomically profiled using 16 S rRNA sequencing. The differential abundance of the ASVs present were determined (Fig. 5a) and analysed using DESeq2. An analysis of how sex influenced the abundance of different microbial families and genera in piglets, regardless of diet, revealed that female piglets exhibited significantly elevated levels of several bacterial families, including Weeksellaceae (*p* < 0.001), Acrobacteraceae (*p* = 0.03), Xanthobacteraceae (*p* = 0.03), and Moraxellaceae (*p* = 0.03) (Fig. 5b). Additionally, the genera *Chrysobacterium* (*p* < 0.001) and *Bradyrhizobium* (*p* = 0.042) were also more abundant in females than in males (Fig. 5c). Further, when comparing sex within the dietary groups, a distinct interaction between sex and diet emerged. In the SP condition, there were 12 genera with significant differences between males and females, including the potentially pathogenic *Eschericia-shigella* (*p* < 0.001), which was more abundant in females, and the generally protective *Bifidobacterium* (*p* < 0.01), which was more abundant in males (Fig. 5d). Similarly, within the HP diet condition, multiple sex differences in bacterial abundance were observed, including a higher abundance of genera associated with disease states, *Arthrobacter* (*p* = 0.025) (Fig. 5e) *Bradyrhizobium* (*p* = 0.003) (Fig. 5f), *Chryseobacterium* (*p* < 0.001) (Fig. 5g), *Paracoccus* (*p* < 0.001) (Fig. 5h), *Lawsonella* (*p* = 0.011) (Fig. 5i), and *Staphylococcus* (*p* < 0.001) (Fig. 5j) in the faecal microbiota of females in comparison to males. Conversely, males exhibited elevated levels of *Pyramidobacter* (*p* = 0.007) (Fig. 5k), butyrate-producing Lachnospiraceae NK4A136 (*p* = 0.007) (Fig. 5l), and the potentially pathogenic *Cloacibacillus* (*p* = 0.016) (Fig. 5m). The presence of more sex differences in the abundance of bacterial genera within each of the diet groups in comparison to when the dietary groups are analysed together.

**Fig. 6:**
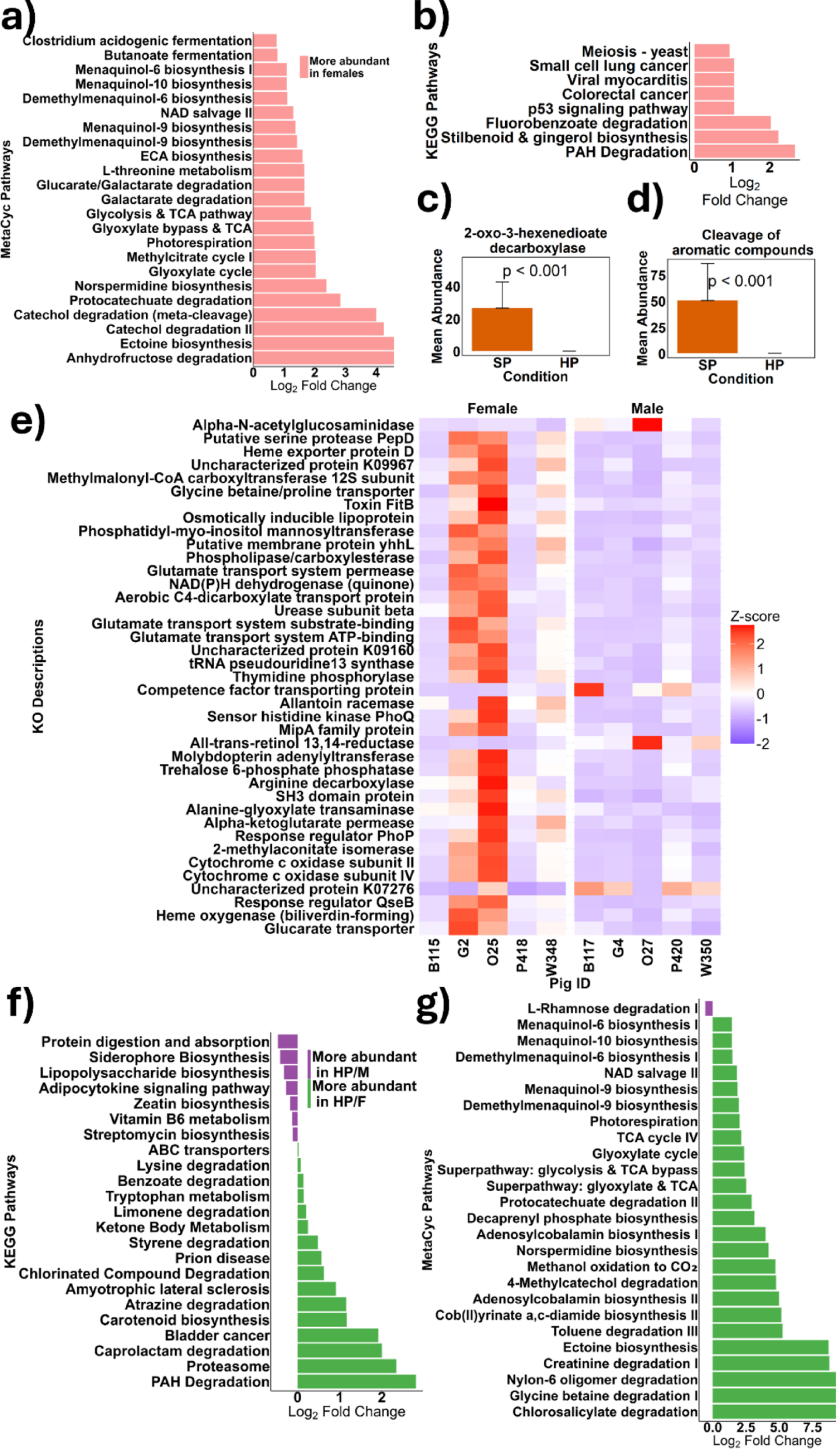
The functional abundance profiles of faecal microbiota in 20 piglets (*n* = 10 males, *n* = 10; females) after consuming either a standard protein (SP; 18% dietary protein) or high protein (HP; 28% dietary protein) diet for 28 days were determined using PICRUSt2. Differences between males and females were evaluated for MetaCyc pathways **a** and KEGG pathways **b**. The effects of dietary protein were assessed usng KEGG orthologs **c** and MetaCyc pathways **d**. Additionally, we analysed the interaction effects between dietary protein intake, and sex, also using KEGG orthologs **e**, KEGG pathways **f** and MetaCyc pathways **g**. Significances were determined using DESeq2 with Benjamini-Hochberg multiple testing corrections and was set at adjusted* p*-value < 0.05

Functional profiles of the microbiome were characterised using KEGG Orthologs (KOs), MetaCyc pathways, and the KEGG Orthology database. Comparative analysis between groups was conducted using DESeq2. Notably, females had a significantly elevated abundance of the MetaCyc pathways involved in carbohydrate fermentation processes such as *Clostridium* acidogenic fermentation and Butanoate fermentation, in comparison to males, whereas males had no upregulated MetaCyc pathways in comparison to females (Fig. 6a). Similarly, females had an increased abundance in KEGG pathways associated with colorectal cancer, lung cancer, and viral myocarditis (Fig. 6b) in comparison to males. Consumption of a high-protein diet resulted in a significantly lower abundance of 2-oxo-3-hexenedioate decarboxylase (*p* < 0.001) (Fig. 6c), an enzyme involved in the degradation of aromatic compounds, and a reduction in the MetaCyc pathway responsible for the cleavage of aromatic compounds (*p* < 0.001) (Fig. 6d) compared to the SP diet, irrespective of sex. Furthermore, females consuming a high-protein diet exhibited 497 KOs that were significantly upregulated compared to males on the same diet, while 99 KOs were more abundant in males than females within the high-protein group. Figure 6e highlights KEGG orthologs with the lowest adjusted *p-*values and a minimum log_2_ fold difference of 1.5 between the HP/F and HP/M groups.

High-protein diets enhanced the potential of the male microbiome to metabolise protein (*p* = 0.042) and produce lipopolysaccharide (*p* = 0.027) more than in females. Conversely, the female microbiome demonstrated upregulation of pathways linked to disease states such as bladder cancer (*p* = 0.043) and prion disease (*p* = 0.02) (Fig. 6f). Additionally, females on the high-protein diet showed an increase in pathways involved in caprolactam degradation (*p* = 0.026), a metabolite formed from the hydrogenation of phenol to cyclohexanone. The MetaCyc pathways also revealed an increased abundance of pathways associated with menaquinol (vitamin K2) production and biosynthesis of cob(II)yrinate a, c-diamide reductase, an enzyme involved in vitamin B12 synthesis, in females compared to males (Fig. 6g).

### Dietary protein increased the production of *p-*cresol in a sex-dependent manner

**Fig. 7:**
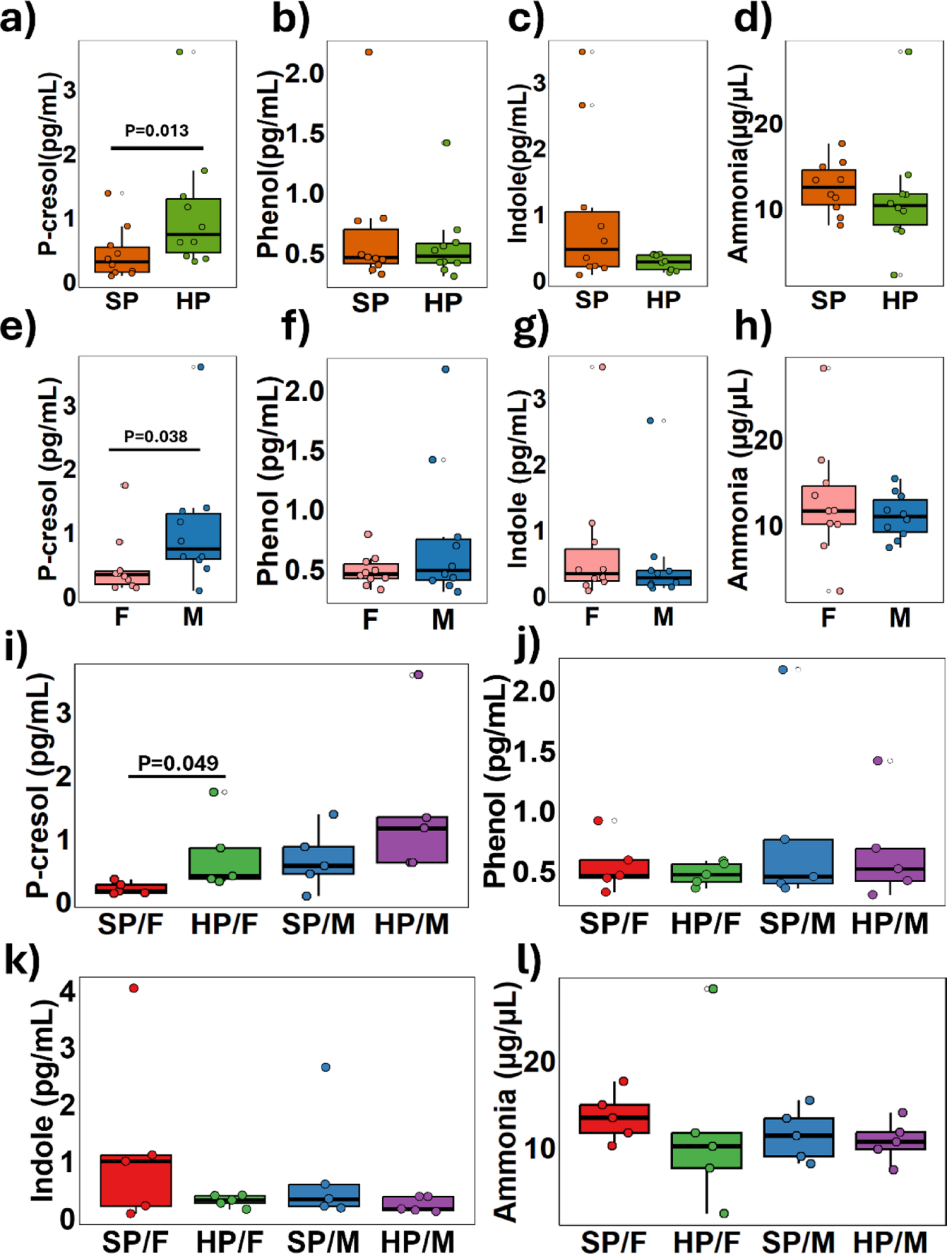
Protein-associated urinary metabolites were excreted from male (M; *n* = 10) and female (F; *n* = 10) piglets consuming either standard protein (SP; 18% dietary protein) or high protein (HP; 28% dietary protein) diets in a 2 × 2 model. The metabolites — *p*-cresol, phenol, indole and ammonia — are produced as a result of fermentation of dietary protein in the gut by the resident microbiota and were quantified using SPME/GC-MS. Differences between dietary protein intake for *p*-cresol **a**, phenol **b**, indole **c** and ammonia **d**; sex differences in *p*-cresol **d** phenol **e**, indole **f**, and ammonia **g**; the interaction between dietary protein intake and sex for *p*-cresol **i**, phenol **j**, indole **k** and ammonia **l** were determined using the Kruskal Wallis test and Wilcoxon rank-sum pairwise comparisons with Benjamini-Hochberg correction. A significant threshold was set at adjusted p-value < 0.05

To measure the end-stage microbial metabolites produced from protein fermentation, urine samples were collected from the bladders of male and female piglets that had consumed either a SP or HP diet for four weeks and analysed using SPME-GCSMS and ammonia assay kits. A significantly higher urinary concentration of *p-*cresol in piglets fed the HP diets compared to those on the SP diets was observed (Fig. 7a) (*p* = 0.013), indicating that excessive dietary protein reached the colon for fermentation by the resident microbiota, which supports the hypothesis. However, no significant differences were noted in other metabolites including phenol (Fig. 7b), indole (Fig. 7c), or ammonia (Fig. 7d).

Urinary *p-*cresol concentrations were significantly higher in males than in females, regardless of their dietary protein intake (Fig. 7e) (*p* = 0.038). However, no differences were found in phenol (Fig. 7f), indole (Fig. 7g) or ammonia (Fig. 7h). Despite the elevated *p-*cresol levels in males, the impact of the HP diets on *p-*cresol was seen in females, not males. Only females on the HP diet exhibited significantly higher levels of urinary *p-*cresol compared to their counterparts on the SP diet (Fig. 7i) (*p* = 0.049), whereas males did not show significant differences in *p-*cresol levels between the two diets. The other metabolites produced by protein fermentation, phenol (Fig. 7j), indole (Fig. 7k), and ammonia (Fig. 7l), were stable across the sex by diet groups.

### The impact of diet and sex on tight cell junction protein expression

**Fig. 8:**
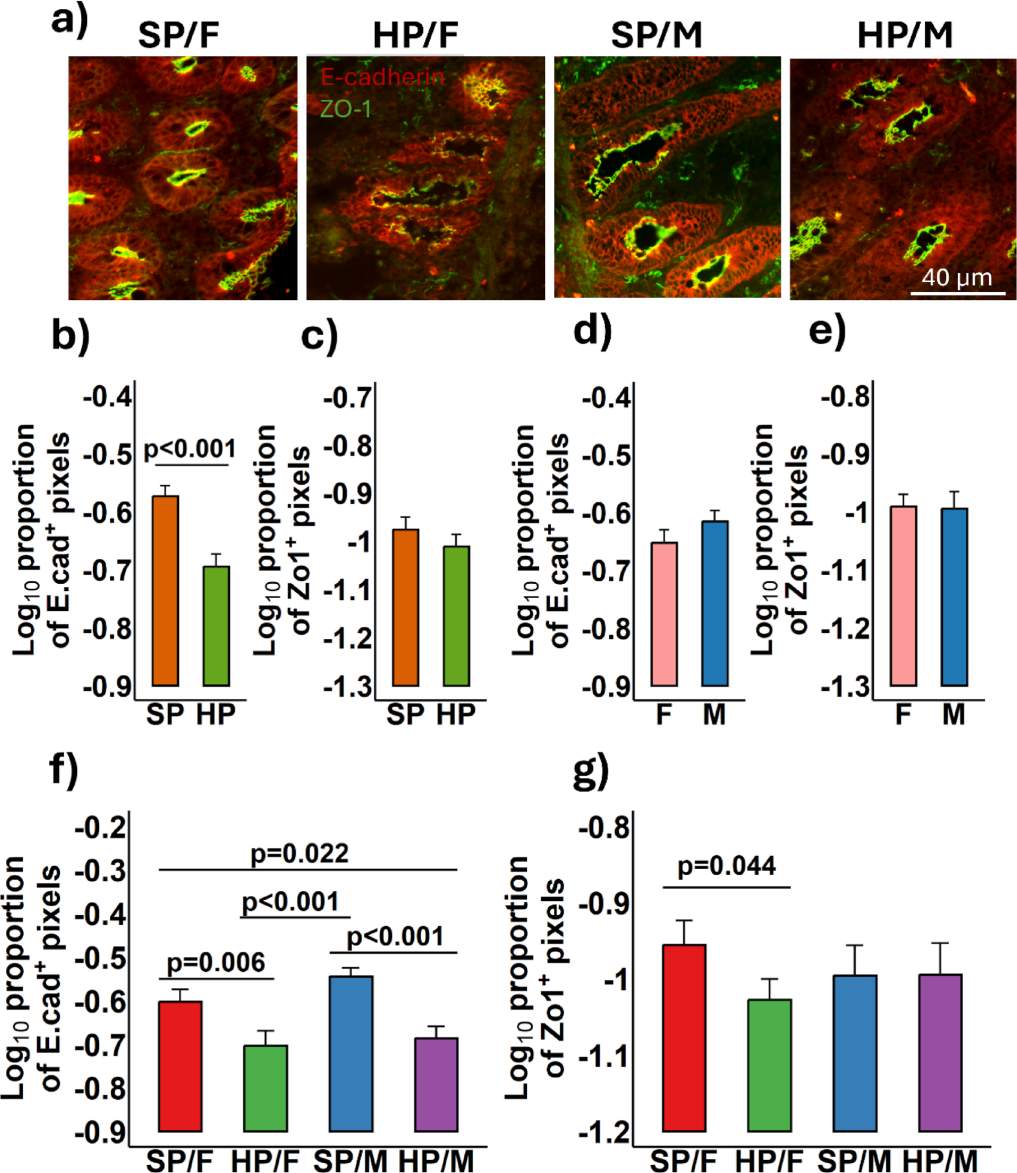
Representational fluorescence immunohistology images of the colon in 56-day old female (F; *n* = 10) and male (M; *n* = 10) piglets fed either a standard protein (SP; 18% dietary protein) or a high protein (HP; 28% dietary protein) diet. Positive staining for E-cadherin (red) and zonula occludens-1 (ZO-1; green) **a**. The Log_10_ proportion of E-cadherin positive pixels **b** and ZO-1 **c** were quantified and compared between dietary groups; differences were also compared between males and females for E-cadherin **d** and ZO-1 **e**; the interaction between diet and sex was also compared for E-cadherin **f** and ZO-1 **g**. Ten individual images were analysed per piglets, with data presented as mean ± SEM and *n* = 5 piglets/sex/treatment group

The expression of proteins located in TCJs in the intestinal epithelium was quantified using fluorescence immunohistology. Representational images of the barrier function-associated proteins, E-cadherin, a component of the adherens junction, and ZO-1 expression are shown in Fig. 8a. Consumption of a HP diet led to a significant reduction in the expression of E-cadherin in colonic tissue in comparison to the SP group (*p* < 0.001) (Fig. 8b), while no significant differences in ZO-1 expression were detected between the dietary groups (*p* = 0.16) (Fig. 8c). These results suggest that HP diets may impair gut barrier function by reducing E-cadherin expression, although they do not appear to affect intercellular ZO-1 when both males and females are analysed together. Similarly, no significant sex differences were observed for either E-cadherin (*p* = 0.15) (Fig. 8d) or ZO-1 expression (*p* = 0.89) (Fig. 8e). The expression of E-cadherin was significantly reduced in both females (*p* = 0.006) and males (*p* < 0.001) consuming the HP diet in comparison to the female and male SP groups (Fig. 8f). However, only females consuming HP diets showed a significantly lower ZO-1 expression (*p* = 0.044) than their same-sex SP group (Fig. 8g).

### Markers of inflammation and bacteria translocation across the epithelium

**Fig. 9:**
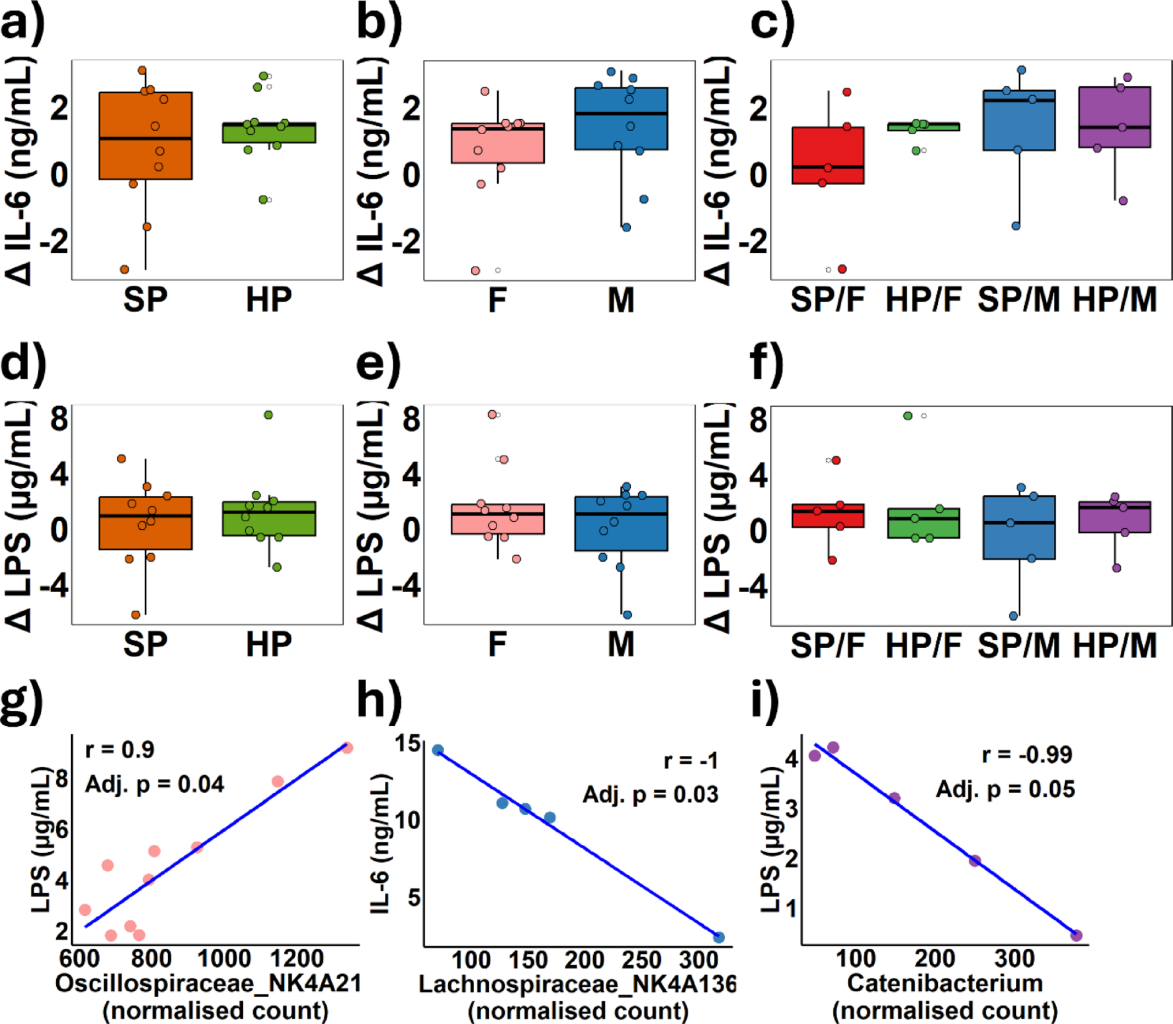
Plasma samples from twenty 56-day-old piglets (*n* = 10 females, F; *n* = 10 males, M), fed either a standard protein (SP; 18% dietary protein) or high protein (HP; 28% dietary protein) diet for 28 days, were analysed for IL-6 and LPS concentrations using ELISA kits. The changes in systemic IL-6 concentration from baseline were calculated and compared for dietary protein groups **a**, sex **b**, and sex by protein group interactions **c**. Similarly, changes in systemic LPS were determined for protein groups **d**, sex **e**, and for sex by protein interactions **f**. Faecal microbiota from the piglets were sequenced using 16 S rRNA sequencing. Microbiota data were normalised using DESeq2, and Shapiro-Wilk normality tests were conducted on each variable. Based on the results of the Shapiro-Wilk test, either Pearson or Spearman correlation coefficients were calculated. Significant correlations after Benjamini-Hochberg correction are presented: females **g**, SP/M **h**, HP/M **I**. statistical significance was set at adjusted* p*-value < 0.05

To assess potential inflammation and intestinal permeability linked to high-protein diet-induced barrier dysfunction, IL-6 and LPS were measured in blood plasma from piglets. The change in IL-6 levels at day 56 from baseline showed no significant differences due to diet (Fig. 9a), sex (Fig. 9b), or any diet-by-sex interactions (Fig. 9c). Similarly, changes in plasma LPS concentrations did not significantly differ between diet groups (Fig. 9d), nor were there differences between females and males (Fig. 9e) or any diet-by sex interactions (Fig. 9f).

### Correlation analyses to determine relationships between microbial genera, *p-*cresol and TCJ proteins

To determine the relationships between the gut microbiota and urinary metabolites (ammonia, indole, phenol, and *p-*cresol), IL-6, LPS, e-cadherin and ZO-1 protein expression, multiple correlation analyses were conducted. Depending on the distribution of the variables, either Pearson or Spearman’s correlation coefficient were used, with the Benjamini-Hochberg correction applied to account for multiple testing. When analysing all piglets together, no significant correlations were observed between any bacterial genera and the other variables. However, in subgroup analyses, a significant positive correlation was identified between *Oscillospiraceae NK4A214* and plasma LPS levels in females (*r* = 0.9, *p* = 0.04) (Fig. 9g). Further subdividing the populations by both sex and dietary protein intake revealed a significant negative correlation between IL-6 and *Lachnospiraceae*_NK4A136 in the SP/M group (*r* = −0.1, *p* = 0.03) (Fig. 9h), as well as a significant negative correlation between *Catenibacterium* and LPS in the HP/M group (*r* = -0.99, *p* = 0.05) (Fig. 9i).

### The influence of high protein diets on the expression of immune-associated proteins in the colonic mucosa

**Fig. 10:**
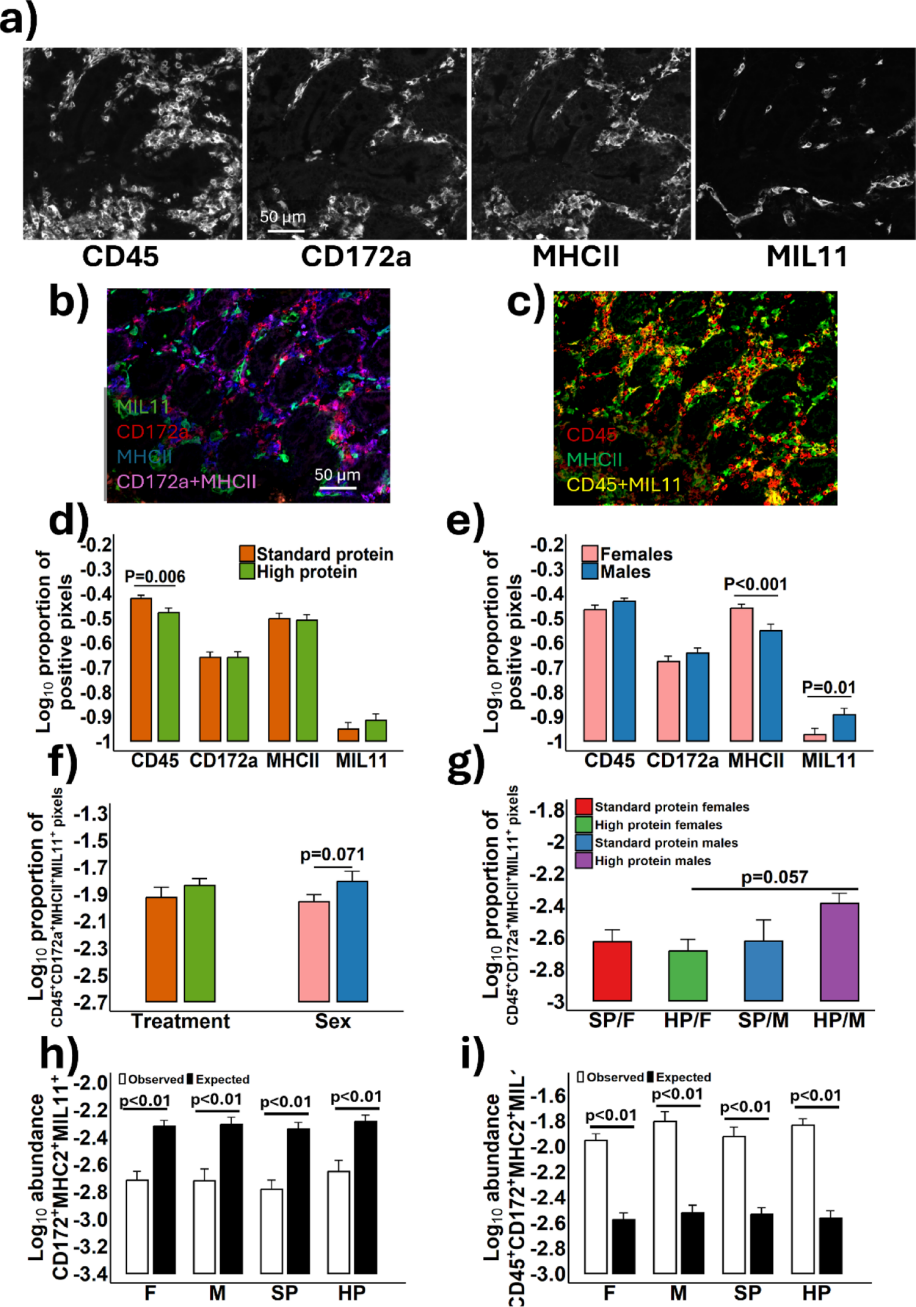
Fluorescence immunohistology representational images depicting the colon in 56-day old female (F; *n* = 10) and male (M; *n* = 10) piglets consuming diets composed of either standard protein (SP; 18% protein) or high protein (HP; 28% protein) **a**. Positive staining is shown for CD45, CD172a, MHCII, and MIL11. Representative images showing co-staining of MIL11, CD172a, and MHCII **b** as well as CD45 and MHCII **c** are also shown. The Log_10_ proportion of positive pixels for each region of interest (lamina propria) were quantified for each protein and compared between dietary protein groups **d** and sex **e**. The co-expression of CD45, CD172a, MHCII, and MIL11 proteins was determined between dietary protein groups and sex **f** as have the interactions between protein intake and sex **g**. The observed proportion of positive pixels for co-expression of CD172, MHCII, and MIL11 **h** and the combination of CD45, CD172a, MHCII and MIL11 **i** were compared against the expected counts of these combinations had the data had random distribution of expression of the proteins. Ten images were analysed per piglet and the data presented are mean ± SEM and *n* = 5 piglets/sex/treatment group

Disruption in gut barrier function may influence immune activation within the lamina propria by enabling increased translocation of antigens through the epithelial barrier. To explore this potential relationship, the expression of immune-associated proteins in the colonic lamina propria of 56-day-old piglets was assessed using fluorescence immunohistology. Figure 10a depicts representative images of positive staining for CD45 (leukocytes), CD172a (Sirp-α, myeloid cells), MHC class II (antigen presenting cells), and capillary endothelium (MIL11). Co-expression was quantified to identify specific cell subsets and assess their potential functional roles within the LP. Representative images show co-expression of MIL11 (green), CD172a (red), and MHCII (blue), with CD172a^+^MILL11^+^ in yellow and CD172a^+^MHCII^+^ in magenta (Fig. 10b). Additionally, co-localisation of MHCII^+^ (green) and CD45^+^ (red) cells shows MHCII^+^CD45^+^ staining (yellow), perhaps indicating antigen-presenting cell activity (Fig. 10c).

Differences in immune-associated protein expression between dietary protein groups, sexes, and interactions between sex and protein intake were quantified. High-protein diets, consumed for four weeks, resulted in significant reductions in the proportion of CD45^+^ cells in the colonic mucosa (log-transformed area; *p* = 0.006) when compared to the SP diet group. However, no significant differences were observed for CD172a^+^, MHCII^+^, or MIL11^+^ expression between dietary groups (Fig. 10d). Females exhibited significantly higher MHCII^+^ staining in the colonic mucosa compared to males (*p* < 0.001), while males had significantly more endothelial cell (MIL11^+^) staining relative to females (*p* = 0.01) (Fig. 10e).

To explore potential interactions between the target immune-associated target proteins, the overlap of positive staining for all four proteins were quantified (CD45^+^, CD172a^+^, MHCII^+^, and MIL11^+^), enabling the potential for antigen presentation between CD45^+^ leukocytes and MHCII^+^ monocytes and endothelial cells in the lamina propria to be assessed. When comparing these interactions across dietary groups, no significant differences were found. However, a trend was observed, perhaps indicating higher levels of antigen presentation in males compared to females (*p* = 0.071) (Fig. 10f) which may have become significant if more animals had been used in the study. Lastly, no significant interactions between sex and dietary protein groups were detected in the expression of CD45, CD172a, MHCII, or MIL11 (data not shown). Nonetheless, a trend towards increased antigen presentation by endothelial cells was noted in the high-protein male group (HP/M) compared to the high-protein female group (HP/F) (*p* = 0.057) (Fig. 10g). Evidence is provided to demonstrate that the co-expression of CD172a, MIL11, MHCII and CD45 observed was significantly higher than that expected from random distribution (*p* < 0.01) (Fig. 10h), whereas when CD45 was omitted (Fig. 10i), the amount of co-staining observed was significantly lower than expected from random distribution, given the amount of single staining measured (*p* < 0.01) for all treatment and sex combinations explored. No other differences were found in the co-expression of immune cells associated proteins; therefore data is not shown.

## Discussion

High-protein diets are prevalent in western societies, but the potential negative consequences of undigested protein reaching the colon and disrupting microbial population dynamics have been largely overlooked. Also, exploration of the microbiota and associated impacts on health and disease are primarily focused on males, or neglect to analyse data by sex. Consequently, crucial sex-based differences could remain undetected, and opportunities to stratify target patient groups for more effectual dietary interventions could be missed. In this study, a sex-balanced, sibling-matched pig model was used to evaluate the effects of increased dietary protein on the microbiota, bacterial-derived metabolite production, barrier function, immunity and inflammation. Our findings show not only microbiota skewing towards a more proteolytic phenotype in response to higher levels of luminal protein availability, but also significant sex-dependent responses. Specifically, females exhibited greater abundances of potentially pathogenic bacteria genera, including *Chryseobacterium*, *Staphylococcus*, and *Paracoccus*, compared to males, which is similar to what has previously been observed in a mouse model [74]. Additionally, on the HP diet, females demonstrated significantly elevated levels of *p-*cresol in urine, a bacterial metabolite linked to increased protein consumption, whereas this increase was not observed in males. Ultimately, consumption of a high-protein diet resulted in a significant reduction in the expression of the barrier integrity-associated protein E-cadherin in both males and females. However, in contrast to males, females exhibited additional significant reductions in the expression of the intercellular TCJ protein ZO-1, in direct response to a high intake of protein.

High-protein diets altered microbial metabolism in the gut, leading to increased *p-*cresol production, which in turn reduced barrier function by decreasing E-cadherin expression in colonic epithelium. This reduction in E-cadherin expression likely occurred because *p-*cresol is a genotoxic agent that disrupts cellular function by reducing cytoplasmic ATP levels through mitochondrial dysfunction [17]. Lower ATP levels impair cell adhesion complexes [17, 75] including adherens junctions [76]. Murine models have shown that HP diets reduced expression of TCJ proteins occludin and ZO-1 [77], and elevated endotoxin levels in the serum [77, 78], indicating a rise in intestinal permeability. Similarly, a study using pigs as models for humans demonstrated that feeding casein directly into the cecum *via* a cannula significantly reduced the expression of ZO-1 and occludin mRNA, and also occludin protein in the gut [79], although none of these studies measured the effect on E-cadherin. Conversely, the current study did not observe changes in ZO-1 expression following a HP diet when both sexes were considered together. This discrepancy may be due to differences in feeding methods. Piglets consumed the protein normally, passing through the digestion process, rather than receiving it directly into the cecum, which means the protein was exposed to more rigorous digestion processes in the upper intestinal tract. Additionally, a combination of four different protein sources were used to increase protein concentration in the HP group, rather than relying solely on casein. Different protein sources can have varied effects on the microbiota, as demonstrated in our previous in vitro model [46]. For example, casein is digested slowly relative to other proteins [80], giving it more time to reach the colon intact and undergo microbial fermentation. Furthermore, individuals that consume HP diets typically do so by consuming a variety of different protein sources, therefore our model may more accurately reflect human dietary patterns. High-protein diets are also consumed with carbohydrates, which may be able to counter some of the negative effects of protein fermentation. Despite observing barrier dysfunction, we did not find significant increases in serum LPS concentrations, which can be used as a proxy measure for intestinal permeability. The reason for this may be that ZO-1 appears to be the primary mediator of barrier function [81], and we saw no changes to ZO-1 expression. As there were no increases in LPS, there were also no significant changes in IL-6 concentration, which may have increased as an inflammatory response to bacterial products passing into the blood stream. However, it is possible that increases in different inflammatory markers, such as TNF-α or faecal calprotectin had increased as a result of the reduction in barrier function proteins, Alternatively, the anti-inflammatory immune phenotype of neonates may have been enough to limit a barrier function-associated immune response in this circumstance.

Irrespective of diet, males and females exhibited significant differences in microbiota composition, *p-*cresol concentrations and MHCII expression in the colonic mucosa. Terminal stool samples showed that, females had greater abundance of *Bradyrhizobium* and *Chryseobacterium* compared to their male siblings. These genera have previously been found in higher concentrations in individuals with diseases associated with gut barrier dysfunction and dysbiosis, including NAFLD [82] and hepatocellular carcinoma [83]. However, since *Bradyrhizobium* is also commonly found in soil, it is possible this was a transient microbe passing through the gut of the piglets after being ingested from the environment. While sex differences in microbiota composition are commonly observed in adults [36, 37], presumably due to the influence of sex hormones [84], it was found that differences emerged in early life when there is minimal hormonal influence on the microbiota. These results are supported by other studies [85, 86] and suggest that other innate physiological functions, which are sexually dimorphic from an early age, are influencing microbiota composition and function. For instance, the immune system significantly influences microbiota composition [87], and in the current study it was found that, irrespective of diet, females had significantly higher MHCII expression in the colonic lamina propria compared to males. This suggests greater levels of antigen-presenting capacity in females, required for immune activation, potentially in response to a higher antigen load. In the current study, males had significantly higher urinary concentrations of *p-*cresol in comparison to females irrespective of dietary protein intake. We previously observed a trend indicating that faecal slurry from healthy human males contained more *p-*cresol than that from human females in the absence of additional protein fermentation [46]. Conversely, other studies have reported higher urinary *p-*cresol levels in autistic female children compared to autistic males [88]. This suggests that, under healthy conditions, females may naturally have lower urinary *p-*cresol concentrations than males.

Interactions between dietary protein and sex in the microbiota, *p-*cresol production and ZO-1 protein expression in the intestinal epithelium were observed. Females on HP diets exhibited significantly greater abundances of *Paracoccus*, a genus which has been found in coronary plaques in individuals with chronic coronary syndrome (CCS) [89], and *Staphylococcus*, which is perhaps unsurprising since it has previously been identified as a protein degrading genera [90]. These differences were associated with significant reductions in ZO-1 protein expression in the colonic epithelium in females, whereas males showed no significant differences in ZO-1 expression between dietary groups. Sex differences in barrier function in response to dietary interventions have been reported previously [91, 92], though this is the first study to demonstrate this effect in vivo in response to HP diets. Evidence suggests that the female epithelial barrier is more vulnerable to perturbation in response to NSAIDs compared to males [47]. One potential mechanism of NSAIDs induced barrier dysfunction could involve mitochondrial dysfunction in combination with elevated ROS levels [93], which is similar to the mechanism by which *p-*cresol disrupts barrier function [75, 94]. Therefore, females may be more susceptible to barrier dysfunction caused by mitochondrial stress and increased ROS production. This may, in part, explain why males did not have reduced barrier function despite having higher *p-*cresol levels when both diet groups were assessed together. The decrease in barrier function in females did not translate to an increase in serum LPS or inflammation, possibly because the reduction in ZO-1 at the TCJ was not severe enough or had not persisted long enough to elevate systemic LPS levels. Furthermore, since these piglets were still young and still in the process of developing immune tolerance to oral antigens [95], increased permeability to bacterial products might have triggered anti-inflammatory responses rather than pro-inflammatory immune responses. If sexually mature piglets had been used in the study the influence of sex hormones on the immune system could have led to more pronounced immune responses. Similarly, it is possible that fermentation of the carbohydrate content of the diet may have countered some of the inflammatory potential caused by the HP diets. Male piglets consuming a HP diet showed a trend toward increased co-expression of CD45^+^CD172a^+^MHCII^+^MIL11^+^ proteins in the lamina propria compared to those on a SP diet, likely in part due to reduced E-cadherin expression. This antigen presenting complex occurs in piglets, but not in pigs, and could therefore contribute to the generation of oral tolerance in young animals [96]. In adults, healthy males typically have more permeable gut barriers than females do [40]. Thus, increased immune tolerance development during early life in response to reduced barrier function may explain why adult males appear to tolerate increased intestinal permeability without triggering elevated inflammatory responses.

Our study was litter-matched, resulting in equivalent genetic contributions to each treatment group and thus, the significant differences we observed were in the same direction and/or of higher magnitude than genetic-based inter-individual differences (which are considerable in outbred pigs and better reflect the human population. Consequently, *n* = 5 was sufficient to identify sex-based responses to HP diets whilst aligning with the 3Rs. However, further confidence in our findings would be achieved if the results were repeated in larger-scale studies. Furthermore, by using a mixture of four proteins to reach the protein level required in the HP group it was unclear what the effects of individual proteins may have on barrier function in vivo. Future research would be required to elucidate this information, although increasing dietary protein using a single source is not reflective of typical diets. Nevertheless, the impacts of dietary protein on the composition of the gut microbiota may be dependent on the origin of the protein; fermentation of fishmeal and soy proteins led to production of significantly more phenol, and indole than milk-casein proteins in a rat model [97]. Furthermore, fermentation of soy protein in the rat caecum generated significantly more acetic acid than fermentation of casein proteins [98]. Consistent with this, we have previously shown that fermenting pea and soya proteins in vitro using faeces from human donors resulted in significantly increased growth of *Roseburia* and *Clostridium coccoides – Eubacterium rectale* groups than the fermentation of egg protein [46]. However, in contrast to these findings, supplementation with both casein and soy proteins did not result in microbiota modifications in overweight adults [99], nor in infants [100]. It is plausible that these inconsistencies are a consequence of differences between species or result from differences in the extraction methods used to obtain the pure proteins used in these studies. For example, hydrolysis is often used to obtain highly purified proteins often used in nutrition trials. However, hydrolysed proteins are highly digestible resulting in only trace amounts reaching the colon. High protein diets are unlikely to be the result of consuming pure protein from a single source, but instead *via* consumption of a range of impure proteins from a diversity of HP containing foods, including meat, dairy and, to a lesser extent, plant-based foods. Such proteins are co-consumed with non-digestible, non-proteins which could also become substrates for colonic gut bacteria, such as dietary fibres which have a beneficial effect in the gut. Perhaps our mix of commonly consumed non-hydrolysed proteins from a variety of sources better reflected human diets and generated more robust, translatable results. *It is acknowledged that the most common animal sources of protein consumed in a typical human diet are meat products*,* yet this study primarily used non-meat animal protein sources to increase the protein content of the HP group. This is because meat-based powdered protein sources (but not fish) have been hydrolysed and are therefore fully digested in the small intestine by the host and do not reach the colon to be fermented by the resident microbiota. Although perhaps a limitation of the model*,* meat protein cannot be extracted using methods which do not hydrolyse the protein.*

In conclusion, HP diets drove shifts in microbial metabolism, and the consequent increases in *p-*cresol production were aligned with reductions in the expression of the adherens junction protein E-cadherin. Additionally, the HP diet had greater impacts on metabolic end-products of protein fermentation in females than in males, resulting in additional reductions in ZO-1 expression in females only. While the current study observed no effect on plasma endotoxin or IL-6 levels in response to diet, this could be due to the limited duration of barrier dysfunction, or the tolerogenic state of immune systems in young mammals. If similar effects of HP diets were to occur in adult humans, they would likely contribute to the chronic inflammation linked to barrier dysfunction. These findings suggest sex-dependent effects of HP diets on the development of disorders associated with barrier dysfunction and chronic inflammation and thus promote adoption of sex-based dietary advice.

## Data Availability

The data that support the finding of this study are openly available in University of Reading Data Archive https://doi.org/10.17864/1947.001381.
